# Efficient Data Gathering Methods in Wireless Sensor Networks Using GBTR Matrix Completion

**DOI:** 10.3390/s16091532

**Published:** 2016-09-21

**Authors:** Donghao Wang, Jiangwen Wan, Zhipeng Nie, Qiang Zhang, Zhijie Fei

**Affiliations:** School of Instrumentation Science and Opto-Electronics Engineering, Beihang University, Beijing 100191, China; dhwang@buaa.edu.cn (D.W.); buaapeng_1417@buaa.edu.cn (Z.N.); youleyuanzq@buaa.edu.cn (Q.Z.); feizhijie@163.com (Z.F.)

**Keywords:** wireless sensor networks, data gathering, compressive sensing, matrix completion, graph based transform, ADMM, A2DM2

## Abstract

To obtain efficient data gathering methods for wireless sensor networks (WSNs), a novel graph based transform regularized (GBTR) matrix completion algorithm is proposed. The graph based transform sparsity of the sensed data is explored, which is also considered as a penalty term in the matrix completion problem. The proposed GBTR-ADMM algorithm utilizes the alternating direction method of multipliers (ADMM) in an iterative procedure to solve the constrained optimization problem. Since the performance of the ADMM method is sensitive to the number of constraints, the GBTR-A2DM2 algorithm obtained to accelerate the convergence of GBTR-ADMM. GBTR-A2DM2 benefits from merging two constraint conditions into one as well as using a restart rule. The theoretical analysis shows the proposed algorithms obtain satisfactory time complexity. Extensive simulation results verify that our proposed algorithms outperform the state of the art algorithms for data collection problems in WSNs in respect to recovery accuracy, convergence rate, and energy consumption.

## 1. Introduction

Wireless sensor networks (WSNs) are composed of large-scale, self-organized sensor nodes, which are capable of sensing, data storage, and communication. WSNs have lots of applications, such as remote environment sensing, industrial automation, smart city, and military monitoring. In practical applications, lots of ordinary nodes are deployed in an unattended mode. These ordinary nodes perform data collection tasks individually and transmit the raw data to the sink node in multi-hop access. Since it is difficult to recharge or replace the limited power supply of ordinary nodes, developing energy efficient data gathering methods is becoming crucial. 

A large number of data collection methods have been proposed to reduce the energy consumption with different levels of data reconstruction precision in the literature [1,2,3]. These obtained data in WSNs possess spatial and temporal correlations, which are intrinsic characteristics of a physical environment. A previous article [1] proposed a clustered aggregation (CAG) algorithm for data collection, which utilizes the spatial and temporal correlations of the sensed data. Pham et al. [2] presented a divide and conquer approximating (DCA) algorithm to reduce power consumption. Since the sensed data require to be transmitted to the sink node in multihop communication, Rosana et al. [3] proposed a novel algorithm to construct spanning trees for efficient data gathering in wireless sensor networks. Unfortunately, data gathering methods in traditional mode have limitations. Firstly, the clustering methods (or the spanning tree construction methods) reflect high computational cost, as well as the dynamic maintaining of clusters (or trees). Secondly, the energy consumption is not balanced since the nodes close to the sink consume more energy.

Compressive Sensing (CS) [4,5,6,7] theory has brought about a new approach for efficient data gathering in WSNs. Since the sensed data have temporal and spatial correlations, they can be sparsely represented in an appropriate transform basis. CS theory states that a small number of linear measurements can accurately reconstruct the sparse signals when the sensing matrix satisfies the restricated isometry property (RIP). Thus, the number of data transmissions for one measurement is largely reduced. Since the high energy-intensive reconstruction algorithm is implemented at the sink node, the computational cost between these ordinary nodes is quite low. Benefiting from the merits of CS, the energy consumption is balanced and reduced for data gathering problem in WSNs. Thus, many papers [8,9,10,11] have been published about efficient data gathering methods based on CS theory in recent years. Luo et al. [8] first proposed to apply compressive sensing for data gathering in WSNs. The idea of the proposed compressive data gathering (CDG) is that the intermediate nodes transmit the weighted sums of father nodes and their own data. In [9], a distributed compressive sampling method was presented. The method is quite efficient since in-network compression is employed, and each node individually determines the transmit scheme to minimize the total number of transmissions. Liu et al. [10] presented the compressed data aggregation (CDA) method to reconstruct the original signals with high precision. Meannwhile, the energy consumption is reduced in comparison with the CDG method. In [11], the authors proposed the compressive data collection (CDC) method to collect data in wireless sensor networks. The scheme reduces the necessary number of measurements, thus the network lifetime is prolonged.

However, the real application of CS in WSNs is difficult. Firstly, CS assumes the data are sparse or could be sparsely represented in a transform basis. Nevertheless, the appropriate sparse matrix basis is not always available. Secondly, the spatial correlation and the temporal correlation cannot be employed together since the sensed data are expressed in the vector form.

As a more efficient data gathering method, matrix completion (MC) [12] considers recovering the incomplete data matrix by observing a small part of the matrix elements. Actually, MC is an extension of the CS theory. In CS, the signals are represented in the vector form, while MC formulates the signals in the matrix form. The sensor data are commonly denoted as matrix, such as the image signals and the video samples. Thus, these two-dimensional signals can be computed more efficiently in the matrix form, although they could be transformed into the form of a vector. In comparison with CS theory, MC do not require to seek a priori sparse basis, and the necessary sampling ratio could be even lower. Since the sensed data collected in WSNs have spatial and temporal correlation, they show low-rank properties. In [13], the singular value thresholding (SVT) algorithm was proposed by approximating the low-rank matrix with a nuclear norm minimization method. To measure large-scale traffic datasets, Roughan et al. [14] proposed the spatial and temporal matrix completion algorithm, which was called the sparsity regularized matrix factorization (SRMF). In [14], intensive analysis of the massive traffic data resulted in the optimal choice of the spatial and temporal constraint matrices. SRMF can be extended to solve various matrix completion based problems, such as data interpolation, and missing matrix elements inference. To further take advantage of the low-rank feature and the short-term stability property of the sensed data, Cheng et al. [15] proposed the STCDG method. The recovery accuracy is improved, and the power consumption is reduced by applying STCDG for data gathering in WSNs.

Actually, the sensor nodes are deployed in a finite area. Therefore, the features of the sensed data are coupled with network topology information. In our analysis, the sensed data are found to be sparse under the graph based transform (GBT) basis. The GBT basis is composed of the eigenvector of the Laplacian matrix when the whole network is represented as a graph. To the best of our knowledge, this is the first time the GBT sparsity has been applied to a matrix completion problem. In consideration of both the GBT sparsity and the low-rank feature of the sensed data, the GBTR-ADMM and the GBTR-A2DM2 algorithm are proposed. The time complexity of our proposed algorithms are also analyzed, which shows that they have a low complexity. Simulation results show our proposed algorithms outperform the state of art algorithms for data collection problems in respect to recovery accuracy, convergence rate, and energy consumption.

The main contributions of the paper are as follows:

(1) The features of sensor datasets are analyzed in consideration of their topology information, which reveals that the data matrix is sparse under the graph based transform.
(2) The graph based transform regularized (GBTR) Matrix Completion problem is formulated. To reconstruct the missing values efficiently, the GBTR by Alternating Direction Method of Multipliers (GBTR-ADMM) algorithm is proposed. Simulation results reveal that GBTR-ADMM outperforms the state of art algorithms in view of the recovery accuracy and the energy consumption.
(3) To accelerate the convergence of GBTR-ADMM, GBTR-A2DM2 algorithm is proposed, which benefits from a restart rule and the fusion of multiple constraints.
(4) The time complexity of our proposed algorithms is analyzed, which shows that the complexity is low.

The structure of the paper is concluded as follows: In Section 2, the problem formulation about matrix completion is given. Section 3 presents the features of the real datasets and the synthesized dataset. The proposed GBTR-ADMM and GBTR-A2DM2 algorithms are expatiated individually in Section 4 and Section 5. Section 6 shows the time complexity of the proposed algorithms. In Section 7, the performances of the proposed algorithms are studied. The conclusions and the future works are summarized in Section 8.

## 2. Problem Formulation

In this section, we introduce the related issues in respect to matrix completion theory. The main notations of the paper are summarized in Table 1.

Suppose there are *N* sensor nodes in the WSNs. Using ${\left\{x^{i}\right\}}_{i=1}^{N}$ denotes the sensor data, where $x^{i}\in {\mathbb{R}}^{M}$ represents a data vector collected by node i in time slot $t_{1},t_{2},\cdots ,t_{m}$. The sample interval is assumed to be equal. Thus, the data matrix $X\in {\mathbb{R}}^{M\times N}$ can be used to represent the sensor data gathered by *N* sensor nodes in *M* time slots. 

In order to reduce energy consumption in resource-constrained WSNs, only a small amount of sensor data is transmitted to the sink node. Let Ω⊂{1,2,⋯,M}×{1,2,⋯,N} denotes the indices of the corresponding observed data of ***X***. Similarly, let $\Omega^{c}$ denotes the indices of omitted value. Let $\pi_{\Omega }$ be the linear projection operator that keeps the entries in Ω invariant and adjusts the entries in $\Omega^{c}$ to zero, that is:

(1) $${\left(\pi_{\Omega }\left(X\right)\right)}_{ij}=\left\{ \begin{array}{ll} X_{ij} & ,\forall \left(i,j\right)\in \Omega \\ 0 & ,\forall \left(i,j\right)\in \Omega^{c} \end{array}\right.$$

Suppose matrix $M\in {\mathbb{R}}^{M\times N}$ is the observed data, which is the incomplete version of matrix ***X*** with entries those outside Ω zeros. That is $\pi_{\Omega }\left(X_{ij}\right)=\pi_{\Omega }\left(M_{ij}\right),\forall \left(i,j\right)\in \Omega$.

Our goal is to reduce the amount of data transmission to the sink node, and to design relevant matrix completion algorithm to reconstruct the original data matrix ***X*** as closely as possible. The observed ratio is defined as:

(2) $$\tau =\frac{\sum_{\left(i,j\right)\in \Omega }\left|\Omega \left(i,j\right)\right|}{MN}$$

Next, the features of the datasets are studied in detail, which would be utilized in our designed algorithms. 

## 3. Exploring the Features of Datasets

**Figure 1:**
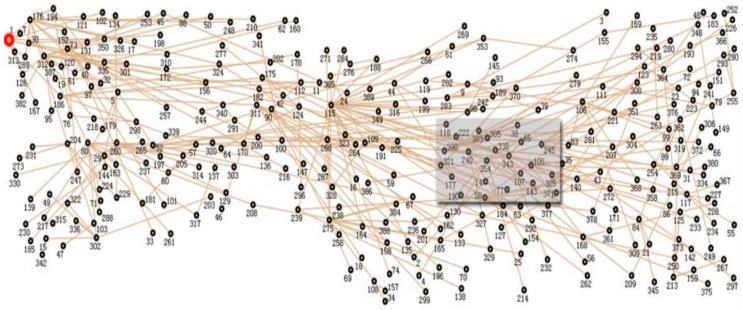
The real deployment topology of GreenOrbs.

**Figure 2:**
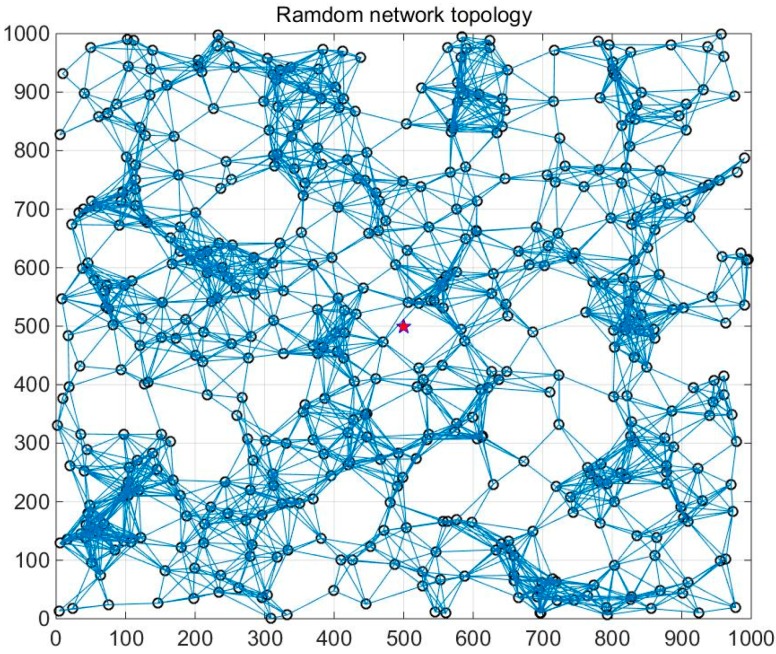
The random topology of synthetized data with 500 nodes in a 1000 m × 1000 m area.

In this subsection, three datasets are utilized for analysis. The first two datasets are gathered by the GreenOrbs [16] system, which is deployed in the forest environment with up to 330 nodes. Its topology is exhibited in Figure 1. We mainly consider the temperature and the humidity data collected between 3 and 5 August 2011. The third dataset is the smooth data generated with a second order Autoregressive (AR) model. In detail, the AR filter $H(z)=\frac{1}{1+a_{1}z^{-1}+a_{2}z^{-2}}$ is used, where $a_{1}$ and $a_{2}$ is predefined as −0.1 and −0.8 individually. Five hundred nodes assigned with the generated data are randomly deployed in a 1000 m × 1000 m area, which is shown in Figure 2. The detailed information about these datasets is given in Table 2.

### 3.1. Low-Rank Property

Since sensor readings have spatial and temporal correlations, the rank r of data matrix ***X*** would be small, such as r≪min(M,N). This low-rank property of the sensor data has been studied in previous papers [14,15,17]. Let rank(X) denote the rank of matrix ***X***. Candes et al. [18] proposed to solve the matrix completion problem by minimizing rank(X) under suitable constraints. However, the minimization problem of rank function cannot be figured out with a global solution in polynomial time because of its non-convexity. Fortunately, the nuclear norm ${\left\|X\right\|}_{*}$, which can be solved using various convex programming methods, is the tightest convex relaxation of the rank function [12]. Also, the relationship between rank function and nuclear norm in matrix completion is similar to the relation between $l_{0}$ norm and the convex $l_{1}$ norm in compressive sensing.

### 3.2. GBT Sparsity

Since these datasets are coupled with their topologies, we construct the graph-based transform (GBT) to sparsely represent them. The sensor network is represented by a graph G=(V,E), which consists of a vertex set *V* (sensor nodes) and an edge set *E* (sensor links). The link e(i,j) is supposed to exist if the Euclidean distance between any two nodes (node i and node j) is smaller than the communication range. The topological graph is mathematically denoted with the adjacent matrix ***A***:

(3) $$A\left(i,j\right)=\left\{ \begin{array}{ll} \;1, & \mathrm{if}\;e(i,j)\mathrm{is}\;\mathrm{an}\;\mathrm{element}\;\mathrm{of}\;E\\ \;0, & \mathrm{otherwise}. \end{array}\right.$$

The degree matrix D is a diagonal matrix, where the diagonal elements D(i,i) denote the number of links connected to node , and D(i,j)=0, ∀i≠j. Thus, the Laplacian matrix can be induced as:

***L*** = ***D*** − ***A*** (4)

Since matrix ***L*** is symmetric, the eigenvalue decomposition can be obtained. Then, the eigenvector of the Laplacian matrix ***L*** constitutes the columns of the graph-based transform matrix, which is denoted as ***Ψ***. Clearly, the GBT matrix ***Ψ*** is orthogonal. Detailed information about GBT basis can be found in [19].

**Figure 3:**
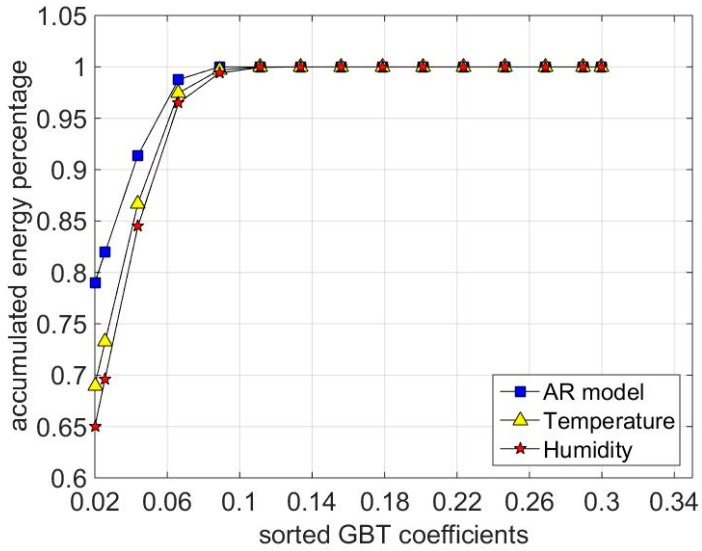
The sorted GBT coefficients of the datasets.

The performance of the GBT matrix as a sparse basis is demonstrated in Figure 3. As can be seen, nearly 10% of the sorted GBT coefficients assemble more the 99% of the energy. Therefore, the matrix ***Ψ***^−1^***X*** is extremely sparse. In other words, the $l_{1}$ norm of matrix ***Ψ***^−1^***X***, represented as ${\left\|\Psi^{-1}X\right\|}_{1}$, is very small.

## 4. The Proposed Optimization Algorithm

Previous matrix completion algorithm is not realistic, as the overfitting problem cannot be avoided when the sampling rate is low. Thus, the recovery error could be large due to the overfitting problem. To obtain exact reconstruction of the missing values, the GBT sparsity and the low-rank property of the data matrix are utilized. Finally, the following optimization problem is formulated:

(5) $$\underset{X}{\min}\left\{{\left\|X\right\|}_{*}+\lambda {\left\|\Psi^{-1}X\right\|}_{1}\right\}\;,\mathrm{subject}\;\mathrm{to}\;\pi_{\Omega }\left(X\right)=\pi_{\Omega }\left(M\right)$$

where λ is the GBT sparsity regularization parameter.

ADMM [20] algorithm can blend the decomposability of dual ascent with an extra variable. Benefitted from the method of multiplier, the algorithm has superior convergence. With an introduced auxiliary variable $W\in {\mathbb{R}}^{M\times N}$, the above problem is rewritten as follows:

(6) $$\underset{X}{\min}\left\{{\left\|X\right\|}_{*}+\lambda {\left\|\Psi^{-1}W\right\|}_{1}\right\}\;,\mathrm{subject}\;\mathrm{to}\;X=W,\pi_{\Omega }\left(W\right)=\pi_{\Omega }\left(M\right)$$

In the following, some prerequisite properties are presented to sever as the foundation to solve the above problem.

**Definition** **1.** *Define the soft-thresholding operator is:*

(7) $$S_{\varepsilon }(\sigma )=\left\{ \begin{array}{ll} \sigma -\varepsilon , & if\;\sigma >\varepsilon ,\\ \sigma +\varepsilon , & if\;\sigma <-\varepsilon ,\\ 0, & otherwise, \end{array}\right.$$

*where ε>0, and the operator can be applied to vectors or matrices in an element-wise manner.*

In consideration of Definition 1, the following helpful theorem is given, as stated in [13].

**Theorem** **1.** *Suppose the Singular Value Decomposition (SVD) of a matrix* $Y\in {\mathbb{R}}^{M\times N}$ *is defined as* $Y=U\Sigma V^{Τ},\;\Sigma =diag\left({\left\{\sigma_{i}\right\}}_{1\leq i\leq \min(M,N)}\right)$ *, where* $U\in {\mathbb{R}}^{M\times r}$ *and* $V\in {\mathbb{R}}^{N\times r}$ *are orthonormal matrix, and* r = rank(Y) *. Then, for any matrix* $Y\in {\mathbb{R}}^{M\times N}$ *and* ∀λ≥0*, the following equations are available:*

(8) $$S_{\lambda }(Y)=\underset{\hat{X}\in {\mathbb{R}}^{M\times N}}{\arg\min}\left\{\frac{1}{2}{{\left\|X-Y\right\|}_{F}}^{2}+\lambda {\left\|X\right\|}_{1}\right\}$$

*and*

(9) $$US_{\lambda }\left(\Sigma \right)V^{Τ}=\underset{\hat{X}\in {\mathbb{R}}^{M\times N}}{\arg\min}\left\{\frac{1}{2}{{\left\|X-Y\right\|}_{F}}^{2}+\lambda {\left\|X\right\|}_{*}\right\}$$

*where* $U\Sigma V^{T}$ *is the SVD of matrix* Y*.*

**Lemma** **1.** *Suppose* $\Psi \in {\mathbb{R}}^{N\times N}$ *is the unit orthogonal real matrix, and* $\Psi^{T}$ *denotes the transpose matrix of* Ψ *. Then, the Frobenius norm of any matrix* A *is invariant under a unitary transformation, that is:*

(10) $${\left\|A\Psi \right\|}_{F}^{2}={\left\|\Psi A\right\|}_{F}^{2}={\left\|A\right\|}_{F}^{2}$$

**Proof.**  Since matrix Ψ is orthogonal, we have $\Psi^{T}\Psi =\Psi \Psi^{T}=I_{N}$. The inverse of matrix Ψ is equal to $\Psi^{T}$. Then, following the definition of trace, we have:

(11) $${\left\|A\Psi \right\|}_{F}^{2}=Tr(\Psi^{T}A^{T}A\Psi )=Tr(A^{T}A\Psi \Psi^{T})=Tr(A^{T}A)={\left\|A\right\|}_{F}^{2}$$

and

(12) $${\left\|\Psi A\right\|}_{F}^{2}\;=\;Tr(A^{T}\Psi^{T}\Psi A)=Tr(A^{T}A)=\;{\left\|A\right\|}_{F}^{2}$$

□

Then, the GBT Regularization by Alternating Direction Method of Multipliers (GBTR-ADMM) is proposed to solve Problem (6). To get rid of the linear constraint in Problem (6), the augmented Lagrangian function is formulated as:

(13) $$L\left(X,Z,W,\beta \right)={\left\|X\right\|}_{*}+\lambda {\left\|\Psi^{-1}W\right\|}_{1}+\langle Z,X-W\rangle +\frac{\beta }{2}{\left\|X-W\right\|}_{F}^{2}$$

where ***Z*** is the Lagrange multiplier and β>0 is the penalty parameter. For a comparative analysis, a fixed parameter β and an adaptive update strategy for optimal β are all studied in the experimental analysis section. GBTR-ADMM updates the variables in a three-step iterative approach under the constraint of fixed β. The augmented Lagrangian function L(X,Z,W,β) is minimized in respect of the variables in a Gauss-Seidel manner. In each step, a single variable is updated by fixing the rest of the variables. By updating the variables alternately, each subproblem is solved with a closed form solution. More specifically, the iterations of GBTR-ADMM are formulated as follows:

Firstly, the variable $X_{k+1}$ is computed with fixed value of $Z_{k}$ and $W_{k}$. Then L(X,Z,W,β) is minimized as follows:

(14) $$\begin{array}{ll} X_{k+1} & =\underset{X}{\arg\min}L\left(X,Z_{k},W_{k},\beta \right)\\ & =\underset{X}{\arg\min}{\left\|X\right\|}_{*}+\lambda {\left\|\Psi^{-1}W_{k}\right\|}_{1}+\langle Z_{k},X-W_{k}\rangle +\frac{\beta }{2}{\left\|X-W_{k}\right\|}_{F}^{2} \end{array}$$

Removing the constant term in Function (14), this function can be rewritten as:

(15) $$\begin{array}{ll} X_{k+1} & =\arg\underset{X}{\min}{\left\|X\right\|}_{*}+\langle Z_{k},X-W_{k}\rangle +\frac{\beta }{2}{\left\|X-W_{k}\right\|}_{F}^{2}\\ & =\underset{X}{\arg\min}{\left\|X\right\|}_{*}+\frac{\beta }{2}{\left\|X-\left(W_{k}-\frac{1}{\beta }Z_{k}\right)\right\|}_{F}^{2} \end{array}$$

Obviously, the above optimization problem has the same form as defined in Theorem 1. Thus, a closed form solution is obtained as follows:

(16) Xk+1=US1β(ΣΡ)VΤ

where U, V, and $\Sigma_{Ρ}$ are obtained from the SVD of matrix Ρ, and Ρ equals to $W_{k}-\frac{1}{\beta }Z_{k}$.

Secondly, the variable $W_{k+1}$ is updated in the choice of default values $X_{k+1}$ and $Z_{k}$. The minimization of L(X,Z,W,β) goes as follows:

(17) $$\begin{array}{ll} W_{k+1} & =\underset{W}{\arg\min}L\left(X_{k+1},Z_{k},W,\beta \right)\\ & =\underset{W}{\arg\min}{\left\|X_{k+1}\right\|}_{*}+\lambda {\left\|\Psi^{-1}W\right\|}_{1}+\langle Z_{k},X_{k+1}-W\rangle +\frac{\beta }{2}{\left\|X_{k+1}-W\right\|}_{F}^{2} \end{array}$$

Ignoring the constant term in this step, we can obtain the following optimization problem:

(18) $$\begin{array}{ll} W_{k+1} & =\underset{W}{\arg\min}L\left(X_{k+1},Z_{k},W,\beta \right)\\ & =\underset{W}{\arg\min}\lambda {\left\|\Psi^{-1}W\right\|}_{1}+\langle Z_{k},X_{k+1}-W_{k}\rangle +\frac{\beta }{2}{\left\|X_{k+1}-W\right\|}_{F}^{2}\\ & =\underset{W}{\arg\min}\lambda {\left\|\Psi^{-1}W\right\|}_{1}+\frac{\beta }{2}{\left\|W-\left(X_{k+1}+\frac{1}{\beta }Z_{k}\right)\right\|}_{F}^{2} \end{array}$$

Taking into consideration of the orthogonal invariance of the Forbenius norm, which is defined in Lemma 1, we obtain the following theorem.

**Theorem** **2.** *The closed form solution of Problem (18) is defined as follows:*

(19) Wk+1=argminWL(Xk+1,Zk,W,β)=ΨSλβ(Ψ−1(Xk+1+1βZk))

**Proof.**  Since matrix $\Psi^{-1}$ is orthogonal, the following equation is obvious in combination with Lemma 1. □

(20) $$\frac{\beta }{2}{\left\|W-\left(X_{k+1}+\frac{1}{\beta }Z_{k}\right)\right\|}_{F}^{2}=\frac{\beta }{2}{\left\|\Psi^{-1}W-\Psi^{-1}\left(X_{k+1}+\frac{1}{\beta }Z_{k}\right)\right\|}_{F}^{2}$$

and defining ***Q*** = ***Ψ*^−1^*W***, we have:

(21) $$\begin{array}{ll} Q_{k+1} & =\underset{Q}{\arg\min}L\left(X_{k+1},Z_{k},Q,\beta \right)\\ & =\underset{Q}{\arg\min}\lambda {\left\|Q\right\|}_{1}+\frac{\beta }{2}{\left\|Q-\Psi^{-1}\left(X_{k+1}+\frac{1}{\beta }Z_{k}\right)\right\|}_{F}^{2} \end{array}$$

By Theorem 1, the closed form solution of the above Problem (21) is obtained as follows:

(22) Qk+1=argminQL(Xk+1,Zk,Q,β)=Sλβ(Ψ−1(Xk+1+1βZk))

Substituting ***Q*** = ***Ψ*^−1^*W*** to Problem (22), then the following closed form solution is available:

(23) Wk+1=argminWL(Xk+1,Zk,W,β)=ΨSλβ(Ψ−1(Xk+1+1βZk))

In view of the second constraint term in Problem (6), the final form of $W_{k+1}$ is defined as:

(24) $$W_{k+1}=\pi_{\Omega }\left(M\right)+\pi_{\Omega^{c}}\left(W_{k+1}\right)$$

Thirdly, with the derived value of $X_{k+1}$ and $W_{k+1}$ in the above two steps, the calculation of Lagrange multiplier is updated as:

(25) $$Z_{k+1}=Z_{k}+\beta \left(X_{k+1}-W_{k+1}\right)$$

The main procedure of GBTR-ADMM is shown in Algorithm 1. Note that the choice of penalty parameter β has high influence on the performance of ADMM algorithm. As it is difficult to choose an optimal value, the adaptive renewal mechanism is preferred in practical application. The performance difference of GBTR-ADMM with varying penalty value is studied in Section 7.1. What is more, the convergence of the ADMM based method is theoretically demonstrated in [20].
**Algorithm 1:** The proposed GBTR-ADMM algorithm.**Initialization:**
$X_{1}=\pi_{\Omega }\left(D\right),W_{1}=X_{1},Z_{1}=X_{1},\beta ,\lambda$**While**
$\left\|X_{k+1}-X_{k}\right\|>\xi$ do1: Xk+1=US1β(ΣΡ)VΤ2: Wk+1=ΨSλβ(Ψ−1(Xk+1−1βZk))In consideration of the constraint in Problem (6)$W_{k+1}=\pi_{\Omega }\left(M\right)+\pi_{\Omega^{c}}\left(W_{k+1}\right)$3: $Z_{k+1}=Z_{k}+\beta \left(X_{k+1}-W_{k+1}\right)$

## 5. The Proposed Method for Accelerated Convergence

The performance of ADMM is highly sensitive to the number of variables and the number of constraints. As is stated in [21,22], more memory is required, and the rate of convergence is reduced with multiple variables constraints. What is more, the convergence property is not proven theoretically when the number of variables is greater than or equal to 3. In optimization Problem (6), these two constraints are considered separately, as shown in Algorithm 1. This may slow down the convergence speed.

In this section, a new approach is proposed to solve Problem (6) with fast constringency speed. Firstly, the two constraints in Problem (6) are merged together in a linear operator. Thus, the convergence rate is accelerated with only one constraint. Then, we introduce the GBT Regularization by accelerated alternating direction method of multipliers (GBTR-A2DM2) As we know, the convergence rate of A2DM2 [23,24] algorithm is O(1k2) while the convergence rate of ADMM (as Algorithm 1) is O(1k).

### 5.1. The Fusion of Two Constraints

In consideration of the two constraints in Problem (6), X=W and $\pi_{\Omega }\left(W\right)=\pi_{\Omega }\left(M\right)$, two linear operators, which are represented as A and ℬ: ${\mathbb{R}}^{M\times N}\to {\mathbb{R}}^{2M\times 2N}$, are defined as follows:

(26) $$A\left(X\right)=(\begin{array}{c} X\\ 0 \end{array}\begin{array}{c} 0\\ 0 \end{array}),ℬ\left(W\right)=(\begin{array}{c} -W\\ \;0 \end{array}\begin{array}{c} \;0\\ \pi_{\Omega }\left(W\right) \end{array}),C=(\begin{array}{c} 0\\ 0 \end{array}\begin{array}{c} \;0\\ \pi_{\Omega }\left(M\right) \end{array})$$

where $C\in {\mathbb{R}}^{2M\times 2N}$ is a constant matrix.

Thus, Problem (6) is reformulated as follows:

(27) $$\underset{X,W}{\min}\left\{{\left\|X\right\|}_{*}+\lambda {\left\|\Psi^{-1}W\right\|}_{1}\right\}\;,\mathrm{subject}\;\mathrm{to}\;A\left(X\right)+ℬ\left(W\right)=C$$

Also, the Lagrange function for the above optimization problem is:

(28) $$L\left(X,Z,W,\beta \right)={\left\|X\right\|}_{*}+\lambda {\left\|\Psi^{-1}W\right\|}_{1}+\langle Z,A\left(X\right)+ℬ\left(W\right)-C\rangle +\frac{\beta }{2}{\left\|A\left(X\right)+ℬ\left(W\right)-C\right\|}_{F}^{2}$$

Similar to Algorithm 1, GBTR-A2DM2 decomposes the minimization of L(X,Z,W,β) into several subproblems. In each subproblem, GBTR-A2DM2 updates a variable keeping in mind that the other variables are fixed. Specifically, the optimization scheme of GBTR-A2DM2 for Problem (28) is resolved in the following steps:

(29) $$\begin{array}{ll} X_{k+1} & =\underset{X}{\arg\min}L\left(X,Z_{k},W_{k},\beta \right)\\ & =\underset{X}{\arg\min}{\left\|X\right\|}_{*}+\langle Z_{k},A\left(X\right)+ℬ\left(W_{k}\right)-C\rangle +\frac{\beta }{2}{\left\|A\left(X\right)+ℬ\left(W_{k}\right)-C\right\|}_{F}^{2}\\ & =\frac{\beta }{2}{\left\|A\left(X\right)+ℬ\left(W\right)-C+\frac{1}{\beta }Z_{k}\right\|}_{F}^{2} \end{array}$$

(30) $$\begin{array}{ll} W_{k+1} & =\underset{W}{\arg\min}L\left(X_{k+1},Z_{k},W,\beta \right)\\ & =\underset{W}{\arg\min}\lambda {\left\|G^{-1}W\right\|}_{1}+\langle Z_{k},A\left(X_{k+1}\right)+ℬ\left(W\right)-C\rangle +\frac{\beta }{2}{\left\|A\left(X_{k+1}\right)+ℬ\left(W\right)-C\right\|}_{F}^{2}\\ & =\underset{W}{\arg\min}\lambda {\left\|G^{-1}W\right\|}_{1}+\frac{\beta }{2}{\left\|A\left(X_{k+1}\right)+ℬ\left(W\right)-C+\frac{1}{\beta }Z_{k}\right\|}_{F}^{2} \end{array}$$

(31) $$Z_{k+1}=Z_{k}+\beta \left(A\left(X_{k+1}\right)+ℬ\left(W_{k+1}\right)-C\right)$$

The pseudocode of GBTR-A2DM2 algorithm is shown in Algorithm 2. Next, we will discuss the accelerated technique of GBTR-A2DM2 with a restarting rule.
**Algorithm 2:** GBTR-A2DM2 algorithm using restarting rule.**Initialization**$W_{0}={\hat{W}}_{0}\in {\mathbb{R}}^{M\times N},Z_{0}={\hat{Z}}_{0}\in {\mathbb{R}}^{M\times N},\tau >0,a_{0}=1,\eta =0.999$**While**
$\left\|X_{k+1}-X_{k}\right\|>\xi$ do1:  Update $X_{k}$ by Equation (29)2:  Update $W_{k}$ by Equation (30)3:  Update $Z_{k}$ by Equation (31)4:  $m_{k}\equiv \frac{1}{\tau }{\left\|Z_{k}-{\hat{Z}}_{k}\right\|}_{F}^{2}+\tau {\left\|ℬ\left(W_{k}-{\hat{W}}_{k}\right)\right\|}_{F}^{2}$**If**
$m_{k}<\eta m_{k-1}$ **Then**5:    $a_{k+1}=\frac{1+\sqrt{1+4a_{k}^{2}}}{2}$6:    ${\hat{W}}_{k+1}=W_{k}+\frac{a_{k-1}}{a_{k+1}}\left(W_{k}-W_{k-1}\right)$7:    ${\hat{Z}}_{k+1}=Z_{k}+\frac{a_{k-1}}{a_{k+1}}\left(Z_{k}-Z_{k-1}\right)$**Else**8:    $a_{k+1}=1,{\hat{W}}_{k+1}=W_{k},{\hat{Z}}_{k+1}=Z_{k}$9:    $m_{k}=\eta^{-1}m_{k-1}$  **End if****End While**


### 5.2. The Accelerated Technique

Since the objective function in Problem (27) is not very convex, the accelerated ADMM method with a restart rule is employed. To determine when to restart the value assignment, the primal error and the dual error are combined:

(32) $$m_{k}\equiv \frac{1}{\tau }{\left\|Z_{k}-{\hat{Z}}_{k}\right\|}_{F}^{2}+\tau {\left\|ℬ\left(W_{k}-{\hat{W}}_{k}\right)\right\|}_{F}^{2}$$

where ${\hat{Z}}_{k}$ and ${\hat{W}}_{k}$ represent the second updated step in iteration steps 6–7 of Algorithm 2. For each iteration, $m_{k}$ is compared with $m_{k-1}$ and if $m_{k}<\eta m_{k-1}$, where η is defined equal to 0.999, the algorithm is accelerated with steps 5–7. Otherwise, the method is restarted in process of steps 8–9. In comparison with GBTR-ADMM, Algorithm 2 has a higher convergence rate. Also, the convergence property of Algorithm 2 is guaranteed by A2DM2 with a restarting rule [23].

## 6. Time Complexity Analysis

In this part, the computational complexity of the proposed algorithms is discussed. The calculation of an inverse matrix cost much, which has the time complexity of O(*n*^3^) (*n* is the dimension of an invertible matrix). Since matrix Ψ is orthogonal, the expensive computation of matrix inversion in our implementation can be substituted by its transposition. Thus, the dominated computational cost of GBTR-ADMM and GBTR-A2DM2 is the execution of matrix SVD in each iteration. As pointed out in [25], the time complexity of SVD operation is O(MN^2^). In our implementation, the famous PROPACK [26] is utilized to perform partial SVD for the proposed algorithms. Since the low-rank property of the objective matrix, it is inefficient to compute the full SVD. To obtain the dominated energy of the objective matrix, only those singular values exceeding than a certain threshold are necessary. The limitation of PROPACK is that it cannot automatically determine the necessary calculations, except for a predefined number. Thus we are supposed to estimate the number of singular values and assign the number to PROPACK in each iteration. 

Suppose $svp^{k}$ is the number of positive singular values of $X_{k}$, and $sv^{k}$ is the number of singular value to be measured at *k*-th iteration. Then, the following updated strategy [27] is used,

(33) $$sv^{k+1}=\left\{ \begin{array}{ll} svp^{k}+1, & if\;svp^{k}<sv^{k}\\ svp^{k}+5, & if\;svp^{k}=sv^{k} \end{array}\right.$$

where the initial estimated value of $sv^{0}$ is 10. Benefiting from the software package PROPACK, the time complexity for a M × N matrix with rank of r is O(*r*MN). Hence, the total time complexity of our proposed algorithms is O(*r*MN). Nevertheless, the state of art algorithms for matrix completion problem [15,17] demand a complexity of O(*r*^2^MN) for each iteration. 

## 7. Performance Evaluation

In this section, we evaluate the performances of GBTR-ADMM and GBTR-A2DM2. The experimental datasets and their topological structures are described in Section 3. Since the proposed algorithms are heavily influenced by several input parameters, it is necessary to choose the optimal parameters to maximize the algorithm performance. With the optimal parameters for GBTR-ADMM and GBTR-A2DM2, the recovery accuracy and the convergence properties are compared with the state of art algorithms. At last, the energy consumption of the proposed algorithms are compared with the state of art data gathering methods for WSNs. Simulation results show that GBTR-ADMM and GBTR-A2DM2 can highly reduce energy consumption in WSNs. Thus, the network lifetime is prolonged.

To measure the performance of the proposed algorithm, the reconstructed data matrix $\hat{X}$ is achieved. Thus, the recovery performance is estimated by the Normalized Mean Absolute Error (NMAE):

(34) $$NMAE=\frac{\sum_{\left(i,j\right)\in \Omega^{c}}\left|\left(\hat{X}\left(i,j\right)-X\left(i,j\right)\right)\right|}{\sum_{\left(i,j\right)\in \Omega^{c}}\left|\Omega^{c}\left(i,j\right)\right|}$$

### 7.1. Parameter Setting

**Figure 4:**
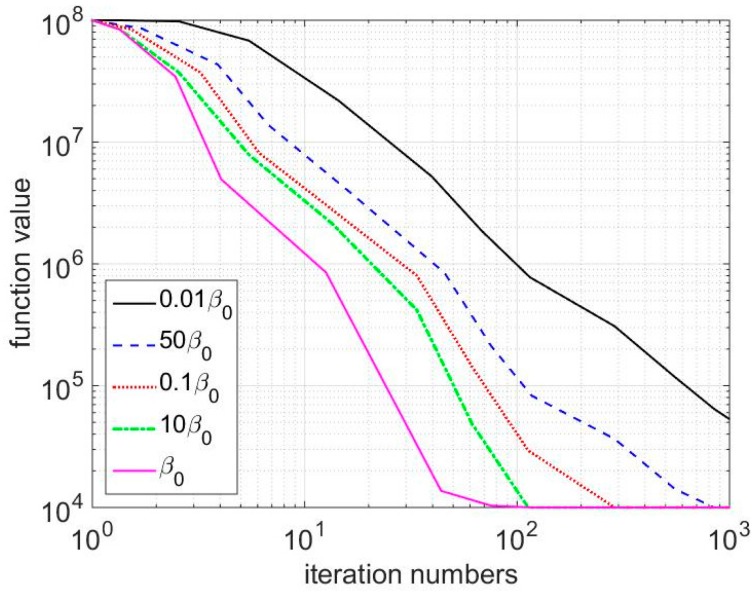
The performance of GBTR-ADMM in respect to different *β*.

In this subsection, the choice of optimal parameters for GBTR-ADMM is discussed. Previous studies have shown that global convergence for ADMM algorithm holds for any fixed *β* > 0. However, different parameter values result in various convergence speeds. Thus, the input values, as listed in Table 3, to Algorithm 1 are selected by experience to obtain the best performance. The performance of GBTR-ADMM is also studied with different parameter values of *β*. The variation of the objective function values of Problem (13) with the increase of iteration numbers is shown in Figure 4. As we can see, *β = β*_0_ achieves the best performance. Meanwhile, the descending speed becomes slower when the choice of parameter *β* is too large or too small. This is because the penalty parameter *β* trades off between minimizing the primal residual and the residual of the dual problem. A large penalty value may drop the primal residual, but at the expense of an increase of the dual residual, and vice versa. 

Figure 4 demonstrates the results in the synthesized datasets, and the optimal chosen value of *β* changes randomly in other datasets. Instead, an adaptive penalty update method is preferred, which is based on previous study [20]. The update strategy is formulated as follows:

(35) $$\beta_{k+1}=\min(\beta_{\max},\rho \beta_{k}),$$

where *β*_max_ denotes the maximum value of the *β_k_*. The value of variable *ρ* is updated as:

(36) $$\rho =\left\{ \begin{array}{l} \rho_{0},\mathrm{if}\frac{\beta_{k}\max\left\{{\left\|X_{k+1}-X_{k}\right\|}_{F}^{2},{\left\|W_{k+1}-W_{k}\right\|}_{F}^{2}\right\}}{{\left\|C\right\|}_{F}}\\ 1,\mathrm{otherwise} \end{array}\right.<\kappa$$

where $\rho_{0}>0$ is a constant and κ is the predefined threshold value. Obviously, when the residual value between ${\left\|X_{k+1}-X_{k}\right\|}_{F}^{2}$ and ${\left\|W_{k+1}-W_{k}\right\|}_{F}^{2}$ is less than the threshold, the value of $\beta_{k+1}$ increases to $\rho_{0}\beta_{k}$. Thus, the convergence speed is improved in this way. 

**Figure 5:**
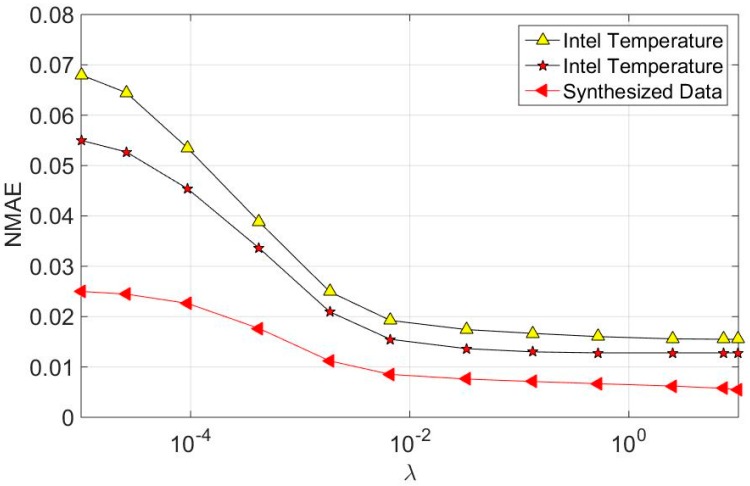
The effect of the sparsity regularization parameter λ.

The effect of the sparsity regularization parameter λ is also analyzed. Figure 5 shows the variation of recovery error with different parameter value. As can be seen, the recovery error is quite large with small value of λ. With the increase of λ, recovery error declines rapidly, and remains stable as λ > 0.01. So, the optimal value for the sparsity regularization parameter λ is set as 0.01 in our experiments. Since similar trends are obtained for GBTR-A2DM2, we just omit it here.

### 7.2. Recovery Accuracy

In this subsection, we compare the recovery accuracy of GBTR-ADMM and GBTR-A2DM2 with the state of art algorithms for matrix completion. The first chosen method is the Spatio-Temporal Compressive Data Collection (STCDG) [15]. The second method is the Compressive Data Collection (CDC) [11]. GBTR-ADMM is considered to solve the optimization Problem (6), while GBTR-A2DM2 is used for the optimization Problem (27) with only one constraint.

Simulations are executed on both the real datasets and the synthesized dataset, which are exploited in detail in Section 3. For each parameter setting of the simulation, the results are averaged for 50 independent trials. 

**Figure 6:**
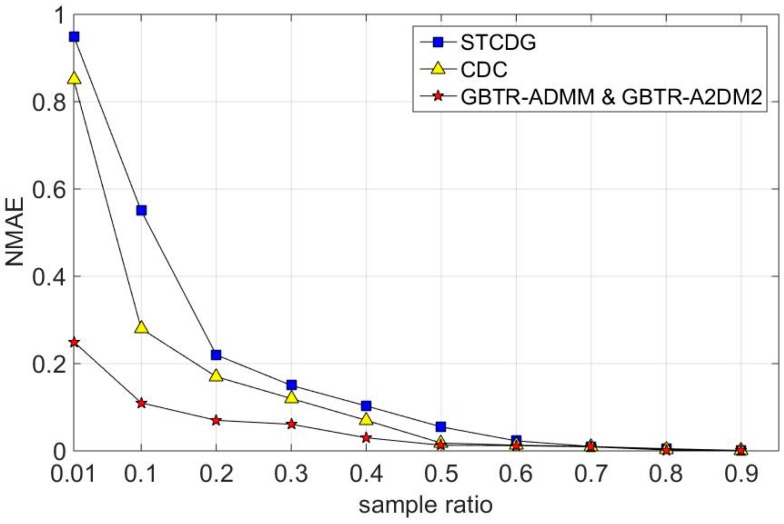
Recovery errors on temperature dataset.

**Figure 7:**
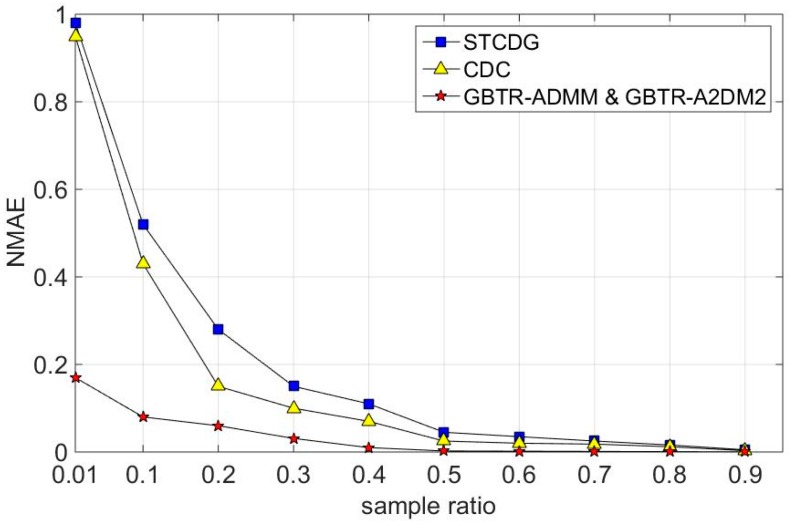
Recovery errors on humidity dataset.

**Figure 8:**
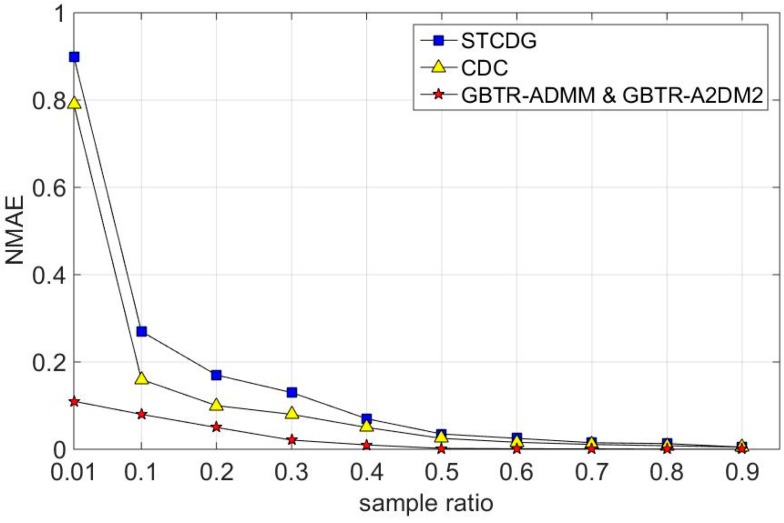
Recovery errors in the synthesized dataset.

Figure 6, Figure 7 and Figure 8 show that our GBTR based methods can reconstruct the missing values with high accuracy. In general, the recovery errors of all reconstruction algorithms decrease rapidly with the increase of the sampling ratio. When the sampling ratio is high enough, all reconstruction methods achieve smaller recovery error. Since our proposed two GBTR based methods are used to solve the same matrix completion problems, their performances are nearly the same. Figure 6 shows the recovery errors on GreenOrbs temperature data. As can been seen, our proposed methods achieve about 25% recovery error while the error of other two algorithms is more than 80%, when the sampling ratio is 1%.

Similar results can be obtained from Figure 7, which is simulated on the humidity dataset. The recovery error of GBTR based methods is still much less than STCDG and CDC when the sampling ratio is small. When the sampling is 1%, GBTR-ADMM and GBTR-A2DM2 can reconstruct the original missing values with recovery errors of less than 20%. Meanwhile, the recovery errors of STCDG and CDC are nearly 100%.

In Figure 8, the experiment results show that GBTR based methods outperform STCDG and CDC by a larger margin. Compared with Figure 6 and Figure 7, Figure 8 shows that GBTR-ADMM and GBTR-A2DM2 achieve the best performance on the synthesized dataset. The recovery error on synthesized dataset is smaller than the real datasets at the same sampling ratio. The reason is that the synthesized data have much better sparsity than the other two real datasets under the GBT basis. 

### 7.3. Convergence Behavior Analysis

**Figure 9:**
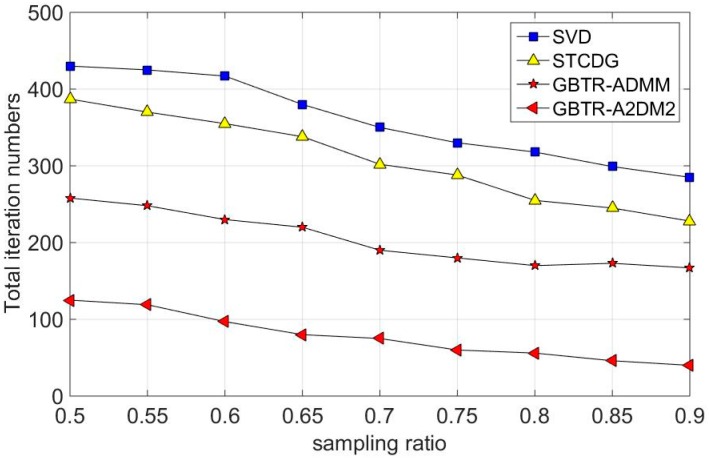
Necessary number of iterations for different algorithms.

In this subsection, the convergence performances are studied in the synthesized dataset. The compared methods are SVD and STCDG. For each method, we set the same stop conditions, where the tolerance error ξ is $10^{-4}$. Figure 9 shows the necessary number of iterations to obtain accurate reconstruction at different sampling ratios. As can be seen from Figure 9, the convergence speed of our proposed two methods surpasses the SVD and STCDG. SVD has the slowest convergence rate of the four methods. Also, STCDG converges faster than SVD. Although the recovery accuracy of GBTR-A2DM2 and GBTR-ADMM behaves similar, as shown in Figure 6, Figure 7 and Figure 8, GBTR-A2DM2 converges much faster than GBTR-ADMM. Note that GBTR-ADMM needs nearly 160 iterations to converge at the sampling ratio 0.9, while only about 40 iterations leads to the convergence of GBTR-A2DM2. Even when the sampling ratio is 0.5, the necessary number of iterations for GBTR-A2DM2 is about 125, which is less than half of GBTR-ADMM.

**Figure 10:**
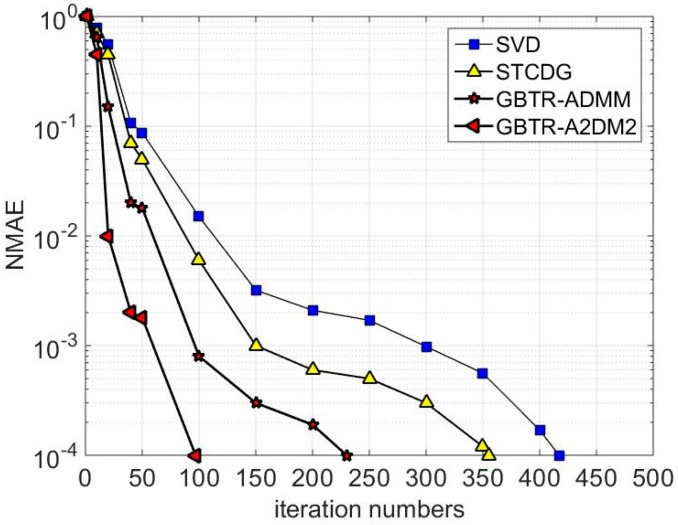
Variation of recovery errors in respect to iteration numbers for different algorithms.

Next, when the sampling ratio is fixed as 0.6, the relative recovery errors of all the compared methods are analyzed. As can be seen from Figure 10, Compared to SVD and STCDG, both GBTR-ADMM and GBTR-A2DM2 gain less error in several iteration processes. Clearly, GBTR-A2DM2 converges much faster, which converges in about 100 iterations. Also, with no more than 250 iterations, both GBTR-ADMM and GBTR-A2DM2 terminate as the relative recovery errors drop below the tolerance error. Meanwhile, the relative errors of SVD and STCDG are about one order of magnitude larger than GBTR-ADMM and GBTR-A2DM2. In general, compared with the state of the art methods for matrix completion, our proposed methods can achieve smaller recovery error at the same number of iterations.

### 7.4. Energy Consumption and Network Lifetime

In this subsection, the energy consumption of the proposed algorithms for data gathering problems is analyzed. Five nodes are randomly deployed in a 1000 m × 1000 m area. The topology is shown in Figure 2, where the sink node is deployed in the center. The data transmission and recovery process are fulfilled in three steps. First, the sink node broadcasts the sampling ratio through the whole network. In our setting, the sampling ratio determines the probability of node to gather data. In the second step, the selected nodes transmit the gathering data to the sink node. Finally, these missing data are reconstructed by implementing GBTR-ADMM and GBTR-A2DM2 at the sink node. The compared methods are CDC and STCDG. In the traditional data gathering method, all sensor nodes are required to transmit their sampling data to the sink node. Thus, the traditional method is selected as a baseline method for comparison.

The energy consumption model in paper [28] is employed in our simulation. The detailed simulation parameters are presented in Table 4. The initial energy for every sensor node is 2 J. Each packet contains 64 bits. The network is supposed to be symmetrical. The energy consumption for one bit transmission, defined as *E_Tx_*, is 100 nJ. Meanwhile, the reception of a packet consumes *E_Rx_* = 120 nJ. $E_{Amp}$ is the unit energy consumption of the power amplifying circuit.

**Figure 11:**
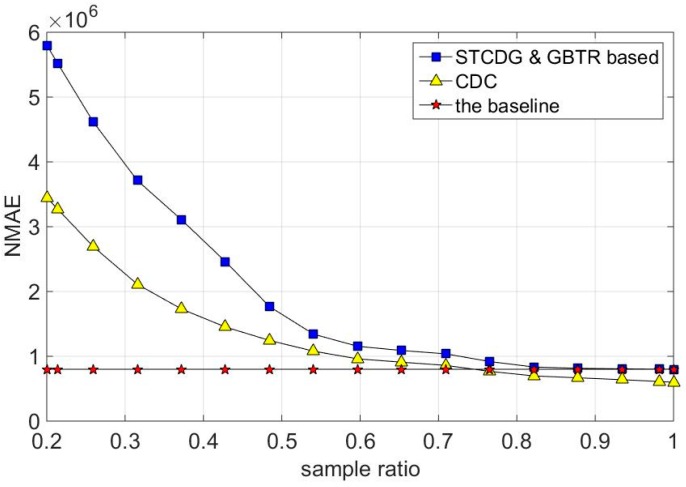
Network lifetime comparison.

The synthesized dataset is exploited in our experiment. The network lifetimes of CDC, STCDG, and our proposed methods are evaluated. Detailed information is revealed in Figure 11. Note that the total energy consumption of the baseline method is relatively constant. That is because the baseline method transmits all the sensor data to the sink node no matter how the sampling ratio varies. To be different, the total power consumptions of CDC, STCDG, and GBTR based methods increase with enlargement of the sampling ratio. The reason is that more sensor data are needed to be transmitted when the sampling ratio increases. Thus, the network lifetime decreases. GBTR-ADMM and GBTR-A2DM2 outperform CDC at the same setting value of sampling ratio. However, the energy consumption of our proposed methods is equal to the baseline method when the sampling radio is exactly 1. Note that the lifetime of CDC is smaller than the baseline when the sampling ratios are higher than 75%. This phenomenon could be explained as per below. In CDC, all sensor nodes transmit the data *M* times, which is the necessary number to reconstruct original signals. At the same time, the ordinary nodes need only one data transmission and necessary relay transmissions for each sampling in the baseline method. Thus, when the sampling ratio is higher than a specific threshold, the total numbers of transmission for CDC is larger than that in the baseline method. In addition, since STCDG and our proposed methods are all based on the matrix completion theory, the curve variations over their lifetime coincide with each other. 

## 8. Conclusions and Future Works

In this paper, the data gathering problem based on Matrix Completion theory is studied. Except for the low-rank property, the sensed data are observed to be sparse under the graph based transform. By taking full advantage of these features, two novel reconstruction algorithms (named GBTR-ADMM and GBTR-A2DM2) are proposed. The time complexity is also analyzed, which shows their complexity is low. Several experiments on both real datasets and synthesized datasets are carried out. The experiment results show that our proposed methods outperform the state of the art algorithm for data gathering problems in WSNs Furthermore, it is observed that GBTR-A2DM2 converges much faster than GBTR-ADMM. For future works, will focus on applying our proposed algorithms to other datasets of real networks, which may exhibit complex topological information other than random networks, such as the scale-free or the small-world networks.

## Figures and Tables

**Table 1:** Summary of notations.

M	Number of time slots
N	Number of sensor nodes
τ	The observed ratio
r	The matrix rank
λ	The GBT sparsity regularization parameter
β	The Lagrange penalty parameter
X	The original data matrix
$\hat{X}$	The reconstructed data matrix
M	The observed data matrix
D	The degree matrix
A	The adjacency matrix
L	The Laplacian matrix
Ψ	The GBT matrix
W	The introduced auxiliary variable
Z	The Lagrange multiplier

**Table 2:** The experimental datasets.

Data Name	Data Types	Selected Data Matrix	Time Interval
GreenOrbs	Temperature	326×500	5 min
GreenOrbs	Humidity	326×500	5 min
Synthesized	AR model	500×500	-

**Table 3:** Input values to Algorithm 1.

Parameter Name	*λ*	*β*
Set Value	0.01	$\beta_{0}=3.0/\min(m,n)$

**Table 4:** Experimental parameters.

Parameter Name	Value
Nodes number	500
Transmission range	100 m
Initial energy	2 J
Data Size	64 bits
$E_{Tx}$	100 nJ/bit
$E_{Rx}$	120 nJ/bit
$E_{Amp}$	0.1 nJ/( bit·m^2^)
